# The Association between Treatment Modality, Lipid Profile, Metabolic Control in Children with Type 1 Diabetes and Celiac Disease—Data from the International Sweet Registry

**DOI:** 10.3390/nu13124473

**Published:** 2021-12-15

**Authors:** Monica Marino, Alexander J. Eckert, Shoshana Tell, Nevena Krnic, Grazyna Deja, Vinni Faber Rasmussen, Raquel Coelho, Sladjana Todorovic, Craig A. Jefferies, Eman Sherif, Carolina Martinez Mateu, Maria Elena Lionetti

**Affiliations:** 1 Department of Pediatrics, Women’s and Children’s Health, Azienda Ospedaliero-Universitaria Ospedali Riuniti Ancona, Marche Polytechnic University, 60121 Ancona, Italy; m.e.lionetti@univpm.it; 2 Institut für Epidemiologie und Medizinische Biometrie, Universität Ulm, ZIBMT, Albert-Einstein-Allee 41, 89081 Ulm, Germany; alexander.eckert@uni-ulm.de; 3 Barbara Davis Center for Childhood Diabetes, University of Colorado Anschutz Medical Campus, Aurora, CO 80045, USA; shoshana.tell@gmail.com; 4 School of Medicine—University Hospital Center Zagreb, University of Zagreb, Diabetology, 10000 Zagreb, Croatia; nevena@krnich.com; 5 Department of Children’s Diabetology, Medical University of Silesia, 40-055 Katowice, Poland; gdeja@sum.edu.pl; 6 Department of Clinical Medicine, Aarhus University, 8200 Aarhus, Denmark; vinni.faber@gmail.com; 7 Department of Pediatric and Adolescents, Randers Region Hospital, 8930 Randers, Denmark; 8 Paediatric Diabetology Department, Protective Association of Diabetics of Portugal (APDP), 1250-189 Lisbon, Portugal; raquel.coelho@apdp.pt; 9 Paediatric Diabetology, Institute of Maternal and Child Health Care of Serbia “Dr Vukan Cupic”, 11000 Belgrade, Serbia; sladjat71@gmail.com; 10 Paediatric Diabetology, Auckland District Health Board (ADHB), Auckland 1051, New Zealand; craigj@adhb.govt.nz; 11 Paediatric Diabetology, Department of Pediatrics, Faculty of Medicine, Ain Shams University, El-Khalifa El-Maamoun, Al Obour, Al Qalyubia Governorate, Cairo 11697, Egypt; eman.sherif@yahoo.com; 12 Pediatrics Buenos Aires, Hospital Garrahan, Buenos Aires C1245 CABA, Argentina; martinezmateu@gmail.com

**Keywords:** type 1 diabetes, continuous subcutaneous insulin infusion, celiac disease, lipid profile, glycemic control

## Abstract

Background and Aims: A higher frequency of dyslipidemia is reported in children with type 1 diabetes (T1D) and celiac disease (CD). Recently, continuous subcutaneous insulin infusion (CSII) has been associated with better lipid profiles in patients with T1D. The aim of this study was to investigate the association between treatment modality and lipid profile, metabolic control, and body mass index (BMI)-SDS in children with both T1D and CD. Methods: Cross-sectional study in children registered in the international SWEET database in November 2020. Inclusion criteria were children (2–18 years) with T1D and CD with available data on treatment modality (CSII and injections therapy, IT), triglyceride, total cholesterol, HDL, LDL, dyslipidemia, HbA1c, and BMI-SDS. Overweight/obesity was defined as > +1 BMI-SDS for age. Data were analyzed by linear and logistical regression models with adjustment for age, gender, and diabetes duration. Results: In total 1009 children with T1D and CD (female 54%, CSII 54%, age 13.9 years ±3.6, diabetes duration 7.2 years ±4.1, HbA1c 7.9% ±1.4) were included. Significant differences between children treated with CSII vs. IT were respectively found; HDL 60.0 mg/dL vs. 57.8 mg/dL, LDL 89.4 mg/dL vs. 94.2 mg/dL, HbA1c 7.7 vs. 8.1%, BMI-SDS 0.4 vs. 0.6, overweight and obesity 17% vs. 26% (all *p* < 0.05). Conclusions: CSII is associated with higher HDL and lower LDL, HbA1c, BMI-SDS, and percentage of overweight and obesity compared with IT in this study. Further prospective studies are required to determine whether CSII improves lipid profile, metabolic control and normalize body weight in children with both T1D and CD.

## 1. Introduction

Celiac disease (CD) is a systemic immune-mediated disorder caused by the ingestion of gluten-containing grains in genetically susceptible persons [1]. While CD prevalence approaches 1% in the general population, [2] it ranges between 1.6% and 9.7% in patients with type 1 diabetes (T1D) worldwide [3], therefore there are a significant number of individuals with both CD and T1D. Despite many prevalence studies, there are few studies about glycemic control, lipid profile, quality of life, microvascular complications, and cardiac risk factors of children with both CD and T1D [4]. 

Individuals with T1D have a higher risk to develop cardiovascular disease compared with the general population [5]. In children with early atherosclerotic signs, dyslipidemia has been found to be present since childhood [6,7]. In addition, it is well-known, that higher level of triglyceride and LDL predict cardiovascular disease [8]. Recent epidemiological studies described increased mortality and higher microvascular complication in individuals with T1D and concomitant CD, suggesting that these patients represent a distinct risk group [9,10]. 

Lipid profile is influenced by gluten-free diet (GFD), which is considered the only available treatment for CD, because of a lower intake of carbohydrates and fiber accompanied by a higher intake of saturated fats, compared with an average diet [11]. A recent systematic review published in 2020 highlighted the association between increased prevalence of weight gain, high blood glucose levels, and a worse lipid profile in celiac patients on a GFD [12]. However, there is a paucity of high-quality evidence on the role of GFD in the context of T1D. A recent large population study showed improved lipid profiles in children and adolescents with T1D treated with continuous subcutaneous insulin infusion (CSII) therapy as compared with injection therapy (IT) [13]. 

The aim of the present study was to investigate the association between treatment modality and lipid profile, metabolic control, and body mass index (BMI) in children with both T1D and CD by analyzing data from the International SWEET Registry.

## 2. Methods

### 2.1. Data Source and Participants 

The SWEET (Better Control in Pediatric and Adolescent Diabetes: Working to Create Centers of Reference) registry is promoted by the International Society for Pediatric and Adolescent Diabetes (ISPAD). The aim of SWEET is to include certified centers for childhood diabetes from all over the world in a community useful for comparisons. The SWEET database currently includes 77,254 participants from 112 diabetes centers worldwide.

As a European Union project, SWEET was approved by the ethical committee at the Auf der Bult Diabetes Centre for Children and Adolescents, Hannover, Germany, wherefrom it is still coordinated, since January 2010, with ethical committee number 848. 

Every participating center is responsible for obtaining appropriate ethical approval and informed consent from children’s parents and guardians and assent from pediatric participants.

This cross-sectional study included children registered in the SWEET database up to July 2020. Inclusion criteria were (a) diagnosis of type 1 diabetes and celiac disease; (b) age between 2 and 18 years; (c) available data on lipid profile (total cholesterol, LDL, HDL, triglyceride) and treatment modality (CSII and IT). Injection therapy includes conventional therapy (1–3 injections a day) and MDI (more than 3 injection a day).

Diagnosis of T1D was performed according to the ISPAD guidelines [14]. CD was defined according to the modified criteria of the ESPGHAN [15]. For each participant we analyzed aggregated data from the most recent documented year, including age, gender, diabetes duration, HbA1c, height, weight, BMI, blood pressure, complications, comorbidities, country of origin and lipid profile. HbA1c was measured locally in each center; to adjust for differences between laboratories, the multiple of the mean method was used to standardize local HbA1c mathematically to the DCCT reference of 20–42 mmol/mol (4–6%). The BMI was calculated from registered height and weight as weight/squared height (kg/m [2]) and converted to BMI-SDS (standard deviation score) using WHO growth curves [16,17]. Blood pressure was assessed according to Fourth Report [18] (“Fourth Report on the Diagnosis, Evaluation, and Treatment of High Blood Pressure in Children and Adolescents”). Participants were divided into the following three groups based on BMI-SDS: normal weight (BMI-SDS 0 to <1.28), overweight (BMI-SDS 1.28 to <1.88) and obese (BMI-SDS ≥ 1.88). Lipid profile assessment included triglycerides, total cholesterol, HDL cholesterol, and LDL cholesterol values. Dyslipidemia was defined in presence of LDL cholesterol ≥100 mg/dL or HDL ≤ 40 mg/dL or total cholesterol ≥ 200 mg/dL. Fasting lipids were measured locally, using standardized, auto-mated instrumentations. 

### 2.2. Statistical Analysis 

All statistical analyses were generated using SAS (Statistical Analysis Software, SAS Institute Inc., Cary, NC, USA) Version 9.4, build TS1M5, on a Windows Server 2016 mainframe. 

Descriptive statistics were performed for all included patients. The results are shown as median with quartiles for continuous variables and as proportions for binary variables.

HbA1c (%), BMI-SDS, total cholesterol, LDL, HDL and triglycerides were analyzed using multivariable linear regression models adjusted for age groups (2–12, >12–18 years), gender and diabetes duration groups (≤5, >5 years). The proportions of individuals with dyslipidemia, overweight and obesity were analyzed using multivariable logistic regression models adjusted for the same variables. Sensitivity analyses were conducted with further adjustment for HbA1c groups (<7.5%, ≥7.5%) and/or BMI-SDS groups (<1.28, ≥1.28). Two-sided *p* < 0.05 indicated a significant difference.

## 3. Results

### 3.1. Descriptive Analysis

**Figure 1:**
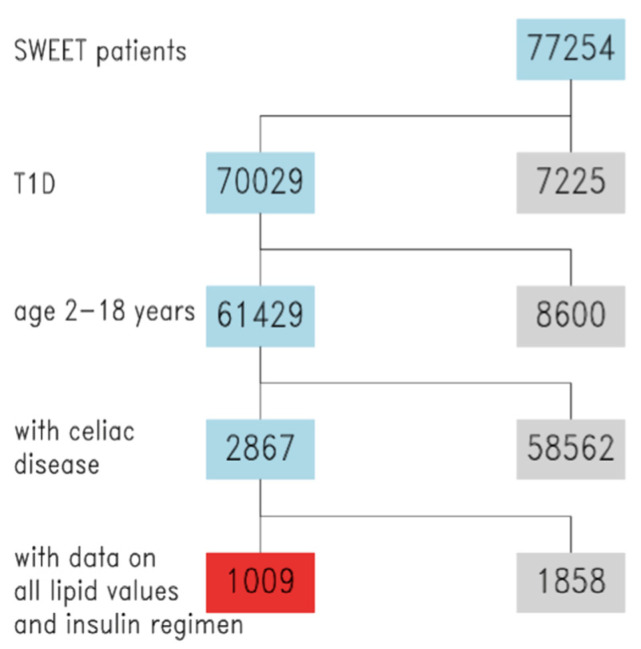
Study flowchart, inclusion of SWEET patients. Blue = inclusion, grey = exclusion, red = final study cohort.

Overall, 62 different centers (44 from Europe, 9 from Asia, Australia and the Middle East, 8 from North and South America) contributed to this analysis. Figure 1 shows the flow-diagram of the inclusion process of patients. 

The final cohort included 1009 children with T1D and CD, 46% male, with mean age 13.9 (range: 11.4–17.2), mean diabetes duration 7.2 years (range: 3.9–10), and mean HbA1c 7.9% (range: 6.9–8.4). CSII therapy was used by 54% of the population, while the others used IT. Demographic features of the study population are shown in Table 1. 

Descriptive analysis, stratified by treatment modality, is reported in Table 2. 

### 3.2. Results from Adjusted Regression Models 

This difference between CSII and IT group was confirmed by linear regression analysis, adjusted for age, gender, and diabetes duration. HbA1c was significantly lower in children treated with CSII as compared with IT [HbA1c 7.7% vs. 8.1%]. In addition, the group of children treated with CSII compared with IT had a significantly lower BMI-SDS [BMI-SDS 0.41 vs. 0.57]. No significant difference in the level of triglycerides or total cholesterol were found. However, a significantly higher level of HDL and a lower level of LDL were observed in children treated with CSII as compared with IT [HDL 60.1 mg/dL vs. 57.6 mg/dL; LDL 89.9 mg/dL vs. 93.6 mg/dL]. All these results are reported in Table 3. 

In addition, the logistic regression models, adjusted for age, gender, and diabetes duration, showed that the percentage of overweight and obesity was significantly lower in children treated with CSII (17% vs. 26%; *p* = 0.0002). There was no significant difference in the percentage of dyslipidemia.

### 3.3. Sensitivity Analysis

After linear regression analysis adjusted for BMI-SDS or HbA1c, the differences between the two groups of treatment are significant. There are no anymore significant differences between the two groups in case of HDL adjusted for BMI, or BMI and HbA1c and LDL adjusted for both BMI and HbA1c. 

## 4. Discussion

Life expectancy in young people with diabetes remains lower than in the general population, despite improvements in glycemic control over the years [19]. Individuals with T1D have a high risk of cardiovascular morbidity and mortality [20]. Subclinical atherosclerotic vascular changes begin in childhood, with several studies showing arterial stiffness and endothelial dysfunction in adolescents with T1D [21,22,23]. LDL-C is a significant predictor of cardiovascular events and mortality in T1D [24]. Each 1 mmol/L (38.7 mg/dL) LDL-C increase is associated with 35–50% more risk of cardiovascular disease, according to a recent study based on the Swedish National Diabetes Registry [24].

It has been established that lowering LDL-C levels, including with lipid-lowering treatment, reduces the risk of developing cardiovascular disease [20]. A reduction of only 1 mmol/dL in LDL-C value is associated with 9% decrease mortality and a 21% decrease in vascular events according to The Cholesterol Treatment Trialists (CCT) study [25]. 

Recently, Kostaria et al. evaluated the effect of CSII on lipid profile in patients with T1D, showing that CSII was associated with improved lipid profiles compared with IT [13]. In particular, LDL-C and non-HDL levels were lower in the CSII group than in the IT group. This finding has been hypothesized to be linked to the improved glycemic control obtained with CSII [6,8,26,27]. 

CD is a co-morbidity of T1D [28]. The only available treatment of CD is the GFD, which consists of the dietary exclusion of grains containing gluten (i.e., wheat, rye, barley, triticale, spelt, and kamut) [11]. A body of evidence has so far suggested that a GFD may be nutritionally unbalanced [28,29,30,31,32,33,34,35,36,37,38,39]. Therefore, adhering to a GFD may further impair the nutritional status, as well as metabolic and lipid profile in patients with both CD and T1D.

Our study shows that the use of CSII is associated with improved glycemic control, BMI-SDS and lipid profile as compared with IT in a large cohort of children and adolescents with both CD and T1D. Firstly, HbA1c was significantly lower in children treated with CSII as compared with IT. The effect of CSII on HbA1c levels in children and adolescents with type 1 diabetes has been largely demonstrated [26,27]. 

Moreover, we found an improved lipid profile in children treated with CSII. Specifically, CSII was significantly associated with higher level of HDL and lower level of LDL-C as compared with MDI, even after adjustment for HbA1c.

It has been hypothesized that by improving the glucose variability and reducing the exposure to periods of hyperinsulinemia, CSII may impact oxidative stress markers and lipid profile [40,41,42]. Children with T1D seem to have higher urinary excretion of 8-iso-PGF2α, F2-isoprostanes formation, than healthy subjects [43,44,45]. These oxidative stress markers enhanced lipid peroxidation and they are correlated with lipid profile alterations [45]. Acute glycemic fluctuations have more effect on oxidative stress than chronic sustained hyperglycemia [46]. CSII treatment is associated with reduced glucose variability because it allows more physiological dosing of insulin [47], and lower total doses of insulin [48]. Indeed, CSII is associated with a lower rate of severe acute complications (severe hypoglycemia and diabetic ketoacidosis) compared with IT, particularly in school-aged children.

Finally, our study shows that in the group of children treated with CSII compared with IT there was a significantly lower BMI-SDS and a significantly lower percentage of overweight and obesity (17% vs. 26%). This finding is of clinical relevance because it is largely known that obesity is a major risk factor for cardiovascular disease development in children and adolescents with type 1 diabetes [47].

The improved anthropometric status of children treated with CSII may be related to a higher diet quality which is directly correlated to better glycemic control in children with T1D and CD [49,50].

A strength of the present study is the fact that the SWEET database comprises a large and heterogeneous, international population, that allows multiple adjustments for major confounding factors, including HbA1c, BMI, age, gender, and diabetes duration, indicating that CSII treatment by itself may contribute to a better lipid profile even in children and adolescents with both T1D and CD.

Limitations of our study are that the SWEET database does not include the start date of the GFD, thus not allowing us to assess the actual adherence of study population to GFD and observe the effect of the duration of GFD on outcome measures evaluated in the present study. 

In addition, the group of IT was significantly higher than the CSII group and had a significantly lower diabetes duration. 

Moreover, we are not able to define whether our findings are due to CSII treatment alone, to sensor-augmented pumps (SAP) therapy or to hybrid closed-loop systems (HCL), advanced hybrid closed loop (AHCL). In addition, the glucose-monitoring strategies were not well-defined in this study, either. Therapy and glucose monitoring strategies affect glycemic control in children with T1D [51].

Lastly, there were not available information on socioeconomic status of study participants. 

## 5. Conclusions

In conclusion, these findings highlight that the choice of treatment methods may have an impact on risk factors for cardiovascular disease in children, particularly those with T1D and CD. 

Further prospective studies are required to investigate the impact of treatment modality and special diet on treatment outcome in children with both T1D and CD and in addition possible underlying pathogenetic mechanisms for this subgroup.

## Figures and Tables

**Table 1:** Demographics of study population, stratified by insulin therapy.

	All Subjects		
	N	Median/Percentage	Lower/Upper Quartile
Demographics			
% males	1009	46	N/A
age (years)	1009	13.9	11.4/17.5
duration of diabetes (years)	1009	7.2	3.9/10.0
height-SDS	1004	0.29	−0.34/0.99
BMI-SDS	1003	0.48	−0.18/1.10
% from Europe	1009	75.2	N/A
% from Asia/Africa ^a^	1009	3.8	N/A
% from Australia ^b^	1009	4.9	N/A
% from North America	1009	12.8	N/A
% from South America	1009	3.4	N/A
Complications and comorbidities			
systolic blood pressure—SDS	917	0.18	−0.44/0.85
systolic blood pressure—SDS	916	0.22	−0.25/0.69
% nephropathy	696	5.2	N/A
% retinopathy	531	4.3	N/A
Diabetes Parameters			
HbA1c (%)	1006	7.86	6.9/8.5
HbA1c (mmol/mol)	1006	62.4	52.3/69.1
total daily insulin dose (U/kg)	920	0.83	0.67/0.99
Lipid parameters			
% Dyslipidemia	1009	42	N/A
TG (mg/dL)	1009	89.4	54.9/105.4
total Chol (mg/dL)	1009	165.0	143.0/182.9
HDL (mg/dL)	1009	58.9	49.5/68.0
LDL (mg/dL)	1009	91.6	73.4/105.0

WHO, World Health Organization, Characteristics of all patients with type 1 diabetes in the study cohort. Data are presented as mean [lower quartile; upper quartile] or proportions., CSII = continuous subcutaneous insulin injection (pump); IT = injections therapy; BMI = body mass index; HDL = high density lipoprotein; LDL = low-density lipoprotein, SDS = standard deviation score (height/BMI according to WHO; blood pressure according to Fourth Report (“Fourth Report on the Diagnosis, Evaluation, and Treatment of High Blood Pressure in Children and Adolescents)). ^a^ Includes Middle East ^b^ Includes New Zealand.

**Table 2:** Descriptive results, stratified by insulin treatment modality.

	Treatment Modality of T1D		
	CSII	IT	*p*-Value
Sex (% males)	44	49	0.502
age (years)	13.3 (10.9–16.9)	14.6 (12.4–17.3)	<0.001
DM duration (years)	7.6 (4.5–10.2)	6.7 (3.4–9.9)	0.002
Height-SDS (WHO)	0.34 (−0.22, 1.07)	0.18 (−0.47, 0.93)	0.033
BMI-SDS (WHO)	0.41 (−0.21, 0.96)	0.57 (−0.14, 1.30)	0.058
HbA1c (%)	7.7 (6.8–8.3)	8.1 (7.0–8.8)	<0.001
daily insulin dose (U/kg)	0.79 (0.65–0.93)	0.88 (0.69–1.05)	<0.001
triglyceride (mg/dL)	88.2 (54–102)	90.8 (55–106.3)	1.000
total cholesterol (mg/dL)	164.3 (143–182)	165.9 (143.1–184)	1.000
HDL (mg/dL)	60.1 (50.3–69.6)	57.6 (47.2–66.1)	0.033
LDL (mg/dL)	89.9 (72–105)	93.6 (74–105.9)	0.499
dyslipidemia (%)	41	43	1.000

T1D = type 1 diabetes; CSII = continuous subcutaneous insulin injection (pump), IT = injections therapy; BMI = body mass index; HDL = high density lipoprotein; LDL = low-density lipoprotein.

**Table 3:** Results on lipid values from adjusted linear regression models.

	Original Model Adjusted for Age, Gender, Diabetes Duration	Model 1 + Adjustment for BMI-SDS	Model 2 + Adjustment for HbA1c	Model 3 + Adjustment for BMI-SDS and HbA1c
Triglycerides [mg/dL]	MDI: 91 [85; 96]	MDI: 90 [84; 96]	MDI: 90 [84; 95]	MDI: 89 [84; 95]
	CSII: 88 [83; 93]	CSII: 89 [84; 94]	CSII: 89 [84; 94]	CSII: 90 [85; 95]
	*p* = 0.5086	*p* = 0.7600	*p* = 0.8880	*p* = 0.8910
Cholesterol [mg/dL]	MDI: 167 [164; 170]	MDI: 166 [163; 169]	MDI: 166 [163; 169]	MDI: 166 [163; 169]
	CSII: 164 [161; 93,166]	CSII: 164 [161; 167]	CSII: 164 [162; 167]	CSII: 165 [162; 167]
	*p* = 0.1305	*p* = 0.2817	*p* = 0.3429	*p* = 0.5488
HDL [mg/dL]	MDI: 58 [56; 59]	MDI: 58 [57; 59]	MDI: 58 [56; 59]	MDI: 58 [57; 59]
	CSII: 60 [59; 61]	CSII: 60 [59; 61]	CSII: 60 [59; 61]	CSII: 60 [59; 61]
	***p* = 0.0157**	*p* = 0.0526	***p* = 0.0192**	*p* = 0.0548
LDL [mg/dL]	MDI: 94 [92; 97]	MDI: 94 [91; 96]	MDI: 94 [91; 96]	MDI: 93 [91; 96]
	CSII: 89 [87; 92]	CSII: 90 [88; 92]	CSII: 90 [87; 92]	CSII: 90 [88; 92]
	***p* = 0.0062**	***p* = 0.0323**	***p* = 0.0224**	*p* = 0.0753

## Abbreviations

AHCL	advanced hybrid closed loop
BMI-SDS	body mass index—standard deviation score
CCT	cholesterol treatment trialists
CD	celiac disease
CSII	continuous subcutaneous insulin infusion
DCCT/EDIC	diabetes control and complications trial/epidemiology of diabetes interventions and complications
ESPGHAN	European Society for Paediatric Gastroenterology Hepatology and Nutrition
GFD	gluten-free diet
HbA1c	glycated hemoglobin
HDL	high-density lipoproteins
ISPAD	International Society of Pediatric and Adolescent Diabetes
IT	injections therapy years
LDL	low-density lipoproteins
MDI	multiple daily injections
SAP	sensor augmented therapy
SAS	statistical analysis system
T1D	type 1 diabetes
WHO	World Health Organisation
